# Development and Evaluation of an Open-Source Software Package “CGITA” for Quantifying Tumor Heterogeneity with Molecular Images

**DOI:** 10.1155/2014/248505

**Published:** 2014-03-17

**Authors:** Yu-Hua Dean Fang, Chien-Yu Lin, Meng-Jung Shih, Hung-Ming Wang, Tsung-Ying Ho, Chun-Ta Liao, Tzu-Chen Yen

**Affiliations:** ^1^Department of Electrical Engineering, College of Engineering, Chang Gung University, Taoyuan 333, Taiwan; ^2^Molecular Imaging Center, Chang Gung Memorial Hospital, Linkou, Taiwan; ^3^Department of Radiation Oncology, Chang Gung Memorial Hospital, Linkou, Taiwan; ^4^Head and Neck Oncology Group, Chang Gung Memorial Hospital and Chang Gung University, Taoyuan 333, Taiwan; ^5^Advanced Research Institute, Institute for Information Industry, Taipei, Taiwan; ^6^Medical Oncology, Chang Gung Memorial Hospital, Linkou, Taiwan; ^7^Department of Nuclear Medicine, Chang Gung Memorial Hospital, No. 5 Fu-Hsin Street, Kwei-Shan, Taoyuan 333, Taiwan; ^8^Otorhinolaryngology, Head and Neck Surgery, Chang Gung Memorial Hospital, Linkou, Taoyuan 333, Taiwan

## Abstract

*Background*. The quantification of tumor heterogeneity with molecular images, by analyzing the local or global variation in the spatial arrangements of pixel intensity with texture analysis, possesses a great clinical potential for treatment planning and prognosis. To address the lack of available software for computing the tumor heterogeneity on the public domain, we develop a software package, namely, Chang-Gung Image Texture Analysis (CGITA) toolbox, and provide it to the research community as a free, open-source project. * Methods*. With a user-friendly graphical interface, CGITA provides users with an easy way to compute more than seventy heterogeneity indices. To test and demonstrate the usefulness of CGITA, we used a small cohort of eighteen locally advanced oral cavity (ORC) cancer patients treated with definitive radiotherapies. * Results*. In our case study of ORC data, we found that more than ten of the current implemented heterogeneity indices outperformed SUV_mean_ for outcome prediction in the ROC analysis with a higher area under curve (AUC). Heterogeneity indices provide a better area under the curve up to 0.9 than the SUV_mean_ and TLG (0.6 and 0.52, resp.). * Conclusions*. CGITA is a free and open-source software package to quantify tumor heterogeneity from molecular images. CGITA is available for free for academic use at http://code.google.com/p/cgita.

## 1. Background

Molecular imaging has become a significant component of patient management in clinical oncology. The importance of extracting quantitative measurements from molecular images has been widely embraced. Recently, there is an increasing interest to quantify the “tumor heterogeneity” from molecular images, especially, PET, as the tumor heterogeneity is an important biomarker for aggressiveness and disease outcome [1–3]. The computation of tumor heterogeneity can be related to texture analysis that refers to numerous mathematical methods to compute quantitative textural features from 2D or 3D images based on the spatial variation of pixel intensity. To properly address the nature of quantification goals of this study, we use the term “heterogeneity index” to denote the calculated tumor heterogeneity in a numerical form. Although there is an emerging enthusiasm in quantification of tumor heterogeneity [4–13], such techniques remain to be evaluated and tested for clinical applications [13].

One existing challenge for investigators interested in testing the usefulness of heterogeneity indices lies in the lack of software available on the public domain. Because texture analysis is a relatively new concept for PET and nuclear medicine community, most software packages offered by vendors do not include functions for such analysis. Commercial third-party software also lacks these functions in general. To address these challenges, we implemented a software package for computing tumor heterogeneity indices and share it with the research community of molecular imaging. This report aims to describe this open-source project of our software package, namely, Chang-Gung Image Texture Analysis (CGITA) toolbox, for quantifying tumor heterogeneity. We will describe its implementation, data flow, and currently supported functions. To evaluate the usefulness of CGITA, we used a cohort of eighteen advanced oral cavity (ORC) cancer patients that were treated with definitive radiotherapy to demonstrate the use of tumor heterogeneity as a biomarker of prognosis assisted by CGITA.

## 2. Methods and Materials

### 2.1. Tumor-Wise and Voxel-Wise Heterogeneity Quantification

The calculation of tumor heterogeneity is implemented in CGITA with two different levels: tumor-wise and voxel-wise heterogeneity. For the former level that generates heterogeneity indices based on the whole delineated tumor, we used the same processing scheme described by Tixier et al. [10]. In brief, a tumor or target volume of tissue is first delineated from the image volume either manually or with automatic segmentation. The intensities of delineated voxels are then redigitized and carried into the mathematical transformation to compute the heterogeneity indices. The second level, on the other hand, computes the heterogeneity indices on a voxel-wise basis. The intensities of surrounding voxels around a specific voxel are used to calculate the heterogeneity indices for this voxel. By repeating the same computation for each voxel, a parametric map can be formed to represent the heterogeneity distribution.

### 2.2. Implementation of CGITA

CGITA was implemented in MATLAB (version 2012a, MathWorks Inc., Natick, MA, USA). It is now distributed over the Internet as an open-source project with two forms of program distribution. For users with a MATLAB license, the MATLAB codes of CGITA are available for them to download, use, and even modify. CGITA is supported and tested on the Windows and Linux platforms. For users without a MATLAB license, a stand-alone CGITA executable is available, although this version does not support the user-defined functions in general. All CGITA functions were implemented in native MATLAB without using compiled C++ functions or MEX files so that cross-platform support can be maximized. The only exception is the dependence of some executable functions in DCMTK [14] on DICOM query and retrieval.

### 2.3. Tumor Delineation

CGITA allows two types of tumor delineation. First, CGITA accepts the volume of interest (VOI) saved as DICOM-RT structures or the VOI drawn and saved with PMOD (PMOD Technologies Ltd, Zurich, Switzerland). Second, the user may use our semiautomatic segmentation functions to delineate the tumor. Currently, the built-in segmentation in CGITA includes a threshold-based region-growing method and a fuzzy C-means method. CGITA allows users to add new segmentation methods as well.

### 2.4. Computational Methods for Whole-Tumor Heterogeneity Indices

We begin by defining what a “heterogeneity index” is. Since the term “heterogeneity” is a general description of mixed composition within an object, there is not a single or specific mathematical definition of heterogeneity. This is why we chose to use the term “heterogeneity index.” Each heterogeneity index indicates the degree of heterogeneity. However, the exact way for computing its value varies from index to index. From the texture analysis point of view, each heterogeneity index is represented by a “textural feature.” We use the term “heterogeneity index” instead of “textural feature” to specifically describe the biological parameter of a tumor that we wish to quantify.

The computation of tumor heterogeneity indices is performed in two steps: the computation of a “parent” matrix and the parameter extraction from this parent matrix. The “parent matrix” refers to a matrix obtained by a numerical transformation that accounts for the spatial arrangement, intensity, and relationship of the voxels contained within the VOI. We have implemented the four parent matrices described in the study by Tixier et al. [10]. We also included an additional four parent matrices: the texture spectrum matrix [15], texture feature coding matrix [16], texture feature coding cooccurrence matrix [16, 17], and neighborhood gray-level dependence matrix [18]. For each of those eight parent matrices, a variable number of heterogeneity indices are calculated.  Table 1 summarizes the currently supported indices in CGITA and their references. At present, there are a total of 72 heterogeneity indices included in CGITA. These heterogeneity indices, in brief, all point to the degree of spatial nonuniformity that directly correlates with tumor heterogeneity in tracer uptake. The difference between individual indices lies in the mathematical computation. For example, the indices computed with voxel-alignment matrix are related to the length of “run,” which is defined as the length of voxels aligned on a line that have the same pixel intensity. Among those indices, for example, the heterogeneity index “long-run emphasis” puts a stronger weighting on the intensities of voxels with long runs. Such an index can be used to measure the tumor heterogeneity by examining the voxels that have the similar tracer uptake and align along the same line.

Some conventional image-derived indices, such as SUV_mean_, SUV_max_, SUL_peak_ [23], and total lesion glycolysis (TLG), are included in these indices based on literatures [10].

### 2.5. Software Validation

The software validation is performed in two different levels. First, we validate the computation of heterogeneity indices against other software packages. Since we do not have access to other in-house software packages for computing the tumor heterogeneity, we are only able to validate some of the conventional indices such as SUV_mean_ and TLG against commercial software PMOD. Tested with clinical PET images, CGITA is able to obtain nearly identical results as PMOD for SUV_mean_ and TLG. The second level of software validation is the software reliability after each update and revision. In order to ensure the software quality, each time a function is added or modified, a set of clinical PET images and its corresponding VOI are kept internally for testing CGITA. Computed heterogeneity indices are generated and compared to historical results, in order to check if the computation remains consistent after software update.

### 2.6. Parametric Imaging

For a given image volume, the parametric image of heterogeneity indices is computed by looping through every voxel and repeating the following steps for each voxel. The user must first choose how many voxels should be used to calculate the parametric image. For example, the user may elect to use a 3 × 3 × 3 cube centered at a specific voxel. The choice of cube size would affect the sharpness of the resulted heterogeneity parametric images. A larger cube size includes more voxels for analysis, potentially improving the heterogeneity accuracy but decreasing the spatial resolution. For a 3 × 3 × 3 cube, the intensities of those twenty-seven voxels are then treated as a delineated volume that is carried into the computation of heterogeneity indices as described in the previous sections. As a result, a heterogeneity index will be computed for the specified heterogeneity index at this given voxel. By looping through all of the voxels, except for those on the edges, a parametric image volume can be formed with the voxel-wise heterogeneity indices.

### 2.7. Evaluation of Heterogeneity Indices for Outcome Prediction in Oral Cavity Cancer Patients

We evaluated the heterogeneity indices implemented in CGITA with patient data from a cohort of ORC patients treated with definitive radiotherapy. These patients are a subgroup of a prospective dose-painting trial, approved by the Institutional Review Board of Chang-Gung Memorial Hospital. This study was conducted in the Linkou and Keelong branches of Chang-Gung Memorial Hospital, from January 2008 to December 2009. A total of 38 nonmetastatic, stage IV ORC patients who were ineligible for radical surgery were included. Informed consent was obtained from every participating subject. Simultaneous integrated boost, intensity-modulated radiation therapy (SIB-IMRT) was used to escalate the irradiation dose within gross tumor volumes to test the treatment efficacy and toxicity. For the quantitative analysis of tumor heterogeneity, we retrieved a uniform treatment group (*n* = 18) who received neoadjuvant chemotherapy followed by concurrent chemoradiation. The group was comprised of 17 males (94.4%) and one female (5.6%), with a median age of 54.2 years (range: 35.9–73.4 years) and a mean age of 53 ± 10.5 years. Most patients had the habits of smoking (*n* = 14, 77.8%), drinking (*n* = 16, 88.9%), and betel-quid chewing (*n* = 16, 88.9%). The preliminary observation was completed in June 2012, and the median follow-up times for all patients were 19.3 months (range: 4.3–50.7) and 38.1 months (range: 30.1–50.7) for survivors. A successful outcome was defined as a state free of disease progression for at least 30 months. Eighteen patients were ultimately classified into the successful group (*n* = 9) and the failed group (*n* = 9). In the successful group, 8 lived without evidence of recurrence, but one died of nondisease-related causes at 16 months after completion of treatment. All other deceased patients died of disease-related events. The pretreatment PET images of all eighteen subjects were used for image analysis.

Each patient in our cohort received a pretreatment FDG-PET/CT scan for staging. Those pretreatment PET/CT scans provided the image sets with which we tested the texture analysis. Fifty minutes after the 370-MBq FDG injection, a whole-body static PET emission scan was acquired on a GE Discovery ST 16 PET/CT (GE Healthcare, Milwaukee, WI) [24] from the skull base to the mid-thigh, with three minutes per bed position. Images were reconstructed with OSEM (ten subsets, four iterations) with pixel spacing of 4.7 mm and 3.3 mm in the transverse and axial directions, respectively. Quantification of the tumor heterogeneity with PET images was performed as follows. First, the tumor contour is delineated by a board-certified nuclear physician in PMOD with a scheme similar to that of the previously reported head and neck tumor delineation [25]. We elected to draw the VOI semiautomatically, since automatic segmentation in ORC patients is generally difficult because some surrounding oral tissues are benign but show a high FDG uptake. After the lesion was first manually outlined from the fused PET/CT images by the nuclear physician, this outlined lesion area was then reviewed to remove benign tissues with high FDG uptake. Once the lesion was outlined, an SUV value of 2.5 was used to delineate the outer contour of the main tumor. Image intensities of the delineated voxels are then used to calculate heterogeneity indices and saved for each patient. Parametric images of heterogeneity indices were calculated for selected subjects.

### 2.8. Performance Evaluation

After the tumor-wise heterogeneity indices were calculated for every subject, the subjects were divided into two groups based on their outcome, with *n* = 9 in each group. A receiver operative characteristics (ROC) curve was plotted for each heterogeneity index independently. We calculated the area under the curve (AUC) from the ROC curves and the optimal sensitivity/specificity for each index. In addition, a Kruskal-Wallis test was performed for each index to evaluate the performance of these metrics [26]. The AUC and the *P* value of the Kruskal-Wallis test calculated from the average intratumor SUV (SUV_mean_) were compared to the AUC and *P* value calculated from each of the other indices.

## 3. Results

**Figure 1:**
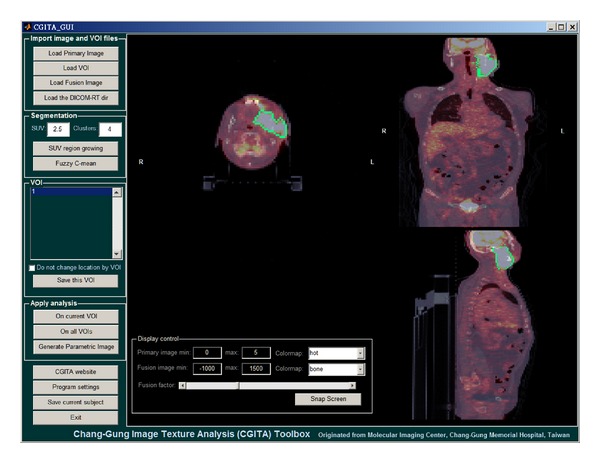
A screen shot of the CGITA program. The CGITA GUI provides users with a simple image display interface that allows users to examine different slices and views. The computation of heterogeneity indices is achieved simply by button clicking. As an open-source project, the current functions and interfaces of CGITA can be customized by users familiar with MATLAB programming. The screen shot here shows a subject with the FDG-PET images fused over CT images.

The appearance of CGITA is shown with a screen shot in Figure 1. Through a graphical user interface (GUI), CGITA's image display interface can be used to view the images and confirm whether the imported VOI aligns properly with the target tissue after importing images and VOIs.  Table 2 summarizes the currently implemented heterogeneity indices in CGITA. The calculated indices, currently totaling 72, can be exported as spreadsheets. In addition to processing one subject at a time using the CGITA GUI, the user may also elect to use the batch mode by processing all subjects automatically without the user input. We tested the batch function on our ORC patient data. On average, each subject takes approximately 30 seconds to process, including image importation and the computation of all 72 features. CGITA is currently hosted at http://code.google.com/p/cgita with both the source code and executables available for download. There is also a user manual for CGITA available at its website. Academic research uses are free of charge. As the hosting service also provides a version control system, users may also participate in this open-source project as developers to contribute new functions to CGITA.

**Figure 2:**
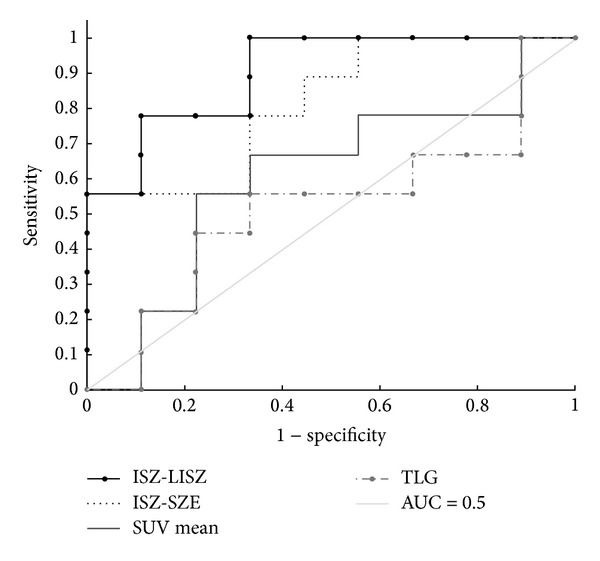
ROC curves of the heterogeneity indices, comparing two of the indices to the conventional metrics. The heterogeneity indices show a higher discriminative power than SUV_mean_ and TLG.

**Figure 3:**
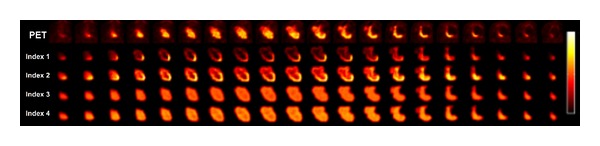
Parametric images of textural features computed from a single patient compared to the original PET image. The PET image, shown in the top row, is displayed between SUVs of zero and twenty. Heterogeneity indices 1 to 4 represent, respectively, the contrast, dissimilarity, entropy, and inverse difference moment calculated from the cooccurrence matrix. Note that the parametric images appear different according to the spatial variation in voxel intensity. Furthermore, different index images display different tumor heterogeneity patterns.

The usefulness of heterogeneity quantification was evaluated with our ORC patient cohort. In terms of outcome prediction, the AUC from ROC analysis was calculated for each heterogeneity index, as was the *P* value for the Kruskal-Wallis test. Out of the total 72 textural features implemented in CGITA, we found that 13 textural features have a higher AUC and 19 have a lower *P* value than the SUV_mean_. The heterogeneity indices with the highest AUC are summarized in Table 3 and compared to conventional markers such as SUV_mean_ and TLG. The conventional markers did not provide satisfactory discriminative power with low AUC (0.6 for SUV_mean_ and 0.52 for TLG) and high *P* value under the Kruskal-Wallis test. On the other hand, some heterogeneity indices stood out as better indicators for prognosis under the current tests. Two indices computed from the intensity-size-zone matrix (ISZ) [21], low-intensity short-zone emphasis (ISZ-LISZE), and short-zone emphasis (ISZ-SZE) showed high AUC (0.9 and 0.81, resp.) and low *P* values (0.004 and 0.024, resp.). Compared to the SUV_mean_, LISZE improved the sensitivity and specificity by 11% and 22%, respectively. Including ISZ-LISZE and ISZ-SZE, six indices computed from the intensity-size-zone matrix provided an AUC over 0.7. The ROC curves of ISZ-LISZE and ISZ-SZE are plotted in Figure 2 along with the ROC curves of SUV_mean_ and TLG. Parametric images based on the textural features of one subject are shown in Figure 3, illustrating a pattern not in the original PET images. Visual inspection revealed that parametric images formed with different heterogeneity indices exhibit various textural patterns.

## 4. Discussion

The search for image-based biomarkers remains an important but challenging aspect of clinical cancer imaging. As imaging technology continues to improve, information extraction from the reconstructed images becomes very important in maximizing the benefit of imaging studies. Recently, the term “radiomics” is proposed to describe the concept of integrating the information extracted from medical images into the proteogenomic and phenotypic information [7, 8]. It is apparent that, for such applications, conventional indices like SUV may not provide sufficient information. Advanced image analysis and information extraction methods become an inevitable component for the concept of radiomics, in order to maximize the amount of information that can be extracted from medical images. Quantification of tumor heterogeneity with texture analysis has been regarded as a promising field by several recent review articles [7, 8, 11, 13]. Recent reports have shown the application of tumor heterogeneity measured with textural features in nonsmall cell lung cancer [4], nasopharyngeal carcinoma [5], cervical cancer [6], peripheral nerve sheath tumors [9], gastrointestinal stromal tumors [12], and esophageal squamous cell carcinoma [27] based on FDG-PET images. Heterogeneity analysis has also been applied to the molecular image analysis of data from animal studies [28–30].

Although texture analysis may be a useful tool to quantify tumor heterogeneity from images, many questions require answers before this concept becomes a clinical standard. The first question arises from the signal and contrast source for different imaging methods. For example, FDG-PET reflects the glucose metabolism of tissues. A tumor that is spatially heterogeneous in cell proliferation may not appear heterogeneous in FDG-PET images, even with perfect resolution, as glucose metabolism may not be directly correlated to the proliferation. On the other hand, the properties of an imaging modality are also critical to texture analysis. The spatial resolution and signal-to-noise ratio (SNR) will both affect the performance of texture analysis. Low spatial resolution degrades the heterogeneity displayed on the acquired image, while high noise will cause a natively homogeneous image to show a high variation in pixel intensity. As a result, the results from texture analysis obtained from clinical PET images should be carefully interpreted and evaluated due to the resolution and noise limitations of PET. Researchers, who believe in the usefulness of quantification for tumor heterogeneity, should take on the responsibility of providing the imaging community with evidence-based studies.

As heterogeneity quantification or texture analysis with molecular images is still a relatively new technology, especially for nuclear medicine community, a free and open-source software package can become a key component for the success of such emerging technologies. Without it, investigators wishing to evaluate heterogeneity indices must develop in-house software, which can be time-consuming and resource limited. A free software package can therefore attract more investigators and allow them to test such new quantification methods on their data with a minimum effort and cost. The other important software characteristic that is much desired is the availability of the source codes to the users. Such efforts for open-source projects in medical imaging have been undertaken by many groups, producing tools such as the kinetic modeling software COMKAT [31, 32] and radiation therapy software CERR [33]. An open-source project has many benefits. It allows the source code to be examined for programming errors. Users with programming abilities may contribute to new functionalities. Most importantly, once the source code is agreed upon by most of the developers, such a software package may become a standardized platform for different groups of researchers to have a common ground for data comparison. A very successful model is the Statistical Parametric Mapping (SPM) [34], which has now become the standard software tool for neuroimaging research as it continues to expand its functionality and user base. CGITA has a long way to go before achieving such maturity like SPM. But with more users and developers being attracted by CGITA, we believe that it has the potential to become the standard software platform for studying the tumor heterogeneity with molecular images. For vendors who wish to develop and test heterogeneity functions in their commercial software, CGITA may also serve as a reference for software verification, thereby accelerating the research and understanding of quantifying tumor heterogeneity.

In brief, CGITA has the following features that may be attractive to different user groups. First, it is easy to use and has a simple GUI. For users without a MATLAB license, a compiled stand-alone executable is also provided on the website. Users without a programming background can easily apply CGITA to their image data. Second, it is open source and allows users to create new functions. Third, it supports more than seventy textural features and its functionalities continue to expand in this regard. Fourth, it has the unique feature of parametric imaging with heterogeneity indices. Finally, CGITA can be executed under a batch mode. The texture analysis of multiple subjects can be performed automatically without user intervention, which is extremely helpful for processing a large amount of data, an unavoidable task for future studies aiming to evaluate the heterogeneity quantification of molecular images. As a result, we believe CGITA will serve as a useful and practical tool for molecular imaging investigators.

A cohort of eighteen ORC patients was used in our study to test our software and demonstrate the potential application of heterogeneity indices. Because the cohort size is small, our intention is not to perform a theoretical study for establishing a standard to stratify advanced ORC patients with textural features. Instead, this case study is a demonstration of how a software package for heterogeneity quantification could be applied to patient data for research purposes. In our report, we chose the task of using heterogeneity indices for prognosis, aiming to test whether heterogeneity indices computed with CGITA might be better discriminators than conventional metrics, such as SUV. Indeed, the results support the claim that textural features may be more informative than SUV in this small-cohort case study. Judging from the ROC analysis, many of the implemented textural features showed better AUC than the AUC with SUV_mean_ in outcome prediction power. Two of these features (ISZ-LISZE, ISZ-SZE) even improve the AUC from 0.6 to 0.81 and 0.9. The sensitivity and specificity have also been improved by heterogeneity indices. Although this study is not aimed to find the theoretical relationship between the heterogeneity indices and disease outcome, we may still speculate the reasons behind such findings. SUV and heterogeneity indices, by nature, represent different physiological and biological mechanisms. In general, SUV represents the “amount” of tracer present in local areas (e.g. SUV_max_) and the whole tumor (SUV_mean_), while heterogeneity indices express the “distribution variation” of tracer activities. The more heterogeneous a tumor is, the more likely a tumor is attempting to differentiate and generating different colonies to survive in its environment, especially during therapy. This may be the underlying reason that enables ISZ-LISZE and ISZ-SZE to improve the AUC from SUV_mean_ because they are capable of capturing the tumor's heterogeneity by emphasizing the tumors with many small “zones,” which is defined as the number of interconnected voxels with the same voxel intensity. As a result, the quantified tumor heterogeneity may serve as a better indicator for tumor aggressiveness which has a direct impact on patients' prognosis and survival. This might explain why heterogeneity indices in general show stronger power for outcome prediction, in our data as well as in the literature [35–37]. However we would also like to point out that, because we have a small patient cohort, the heterogeneity indices that are found with the best performance need to be further validated with a larger amount of data. Further validation is necessary in the future.

The image quality is also an important factor for heterogeneity quantification that requires further study and validation, especially in the spatial resolution and SNR. In our study, we used a PET camera with a spatial resolution of about 6 mm and system sensitivity of about 0.7% [24]. Current state-of-art cameras could achieve a higher spatial resolution of about 4 mm and system sensitivity of 0.9% [38]. Improvement on the spatial resolution with new cameras and image SNR by a higher tracer dose undoubtedly will increase the accuracy for heterogeneity quantification. However, the minimum requirement of image quality for a specific disease remains to be further studied. In our study, since the tumor size is generally large in our cohort with an average volume of 107 mL, a spatial resolution of 6 mm is probably sufficient. Similarly, since the tracer uptake is fairly high with SUV_mean_ above five, the SNR should not be a concern under the standard injection dose of 370 MBq of FDG. With our current data, it is not feasible to determine the smallest tumor or the worst noise level that can still produce accurate tumor heterogeneity quantification. Such studies may require animal or phantom studies, in which the injected dose and image acquisition mode can be more freely modified and tested. CGITA may facilitate such testing on the software side. The exact determination of the spatial resolution and system sensitivity requirements awaits future investigation.

We are not the first group to propose the parametric imaging of heterogeneity indices. For example, a previous report has demonstrated such techniques applied to MRI for lesion segmentation purposes [39]. However, to our knowledge, we are the first group to implement this function as part of an open-source project for quantifying the tumor heterogeneity of medical imaging. We tested this functionality in our patient cohort and were able to obtain heterogeneity parametric images. Unfortunately, we do not possess in vitro images of tumor heterogeneity with which our parametric images are compared because our cohort did not undergo surgical dissection of the tumor. However, it is still quite interesting to examine these images, as shown in Figure 3. For example, in the cooccurrence-contrast images, a hot spot is shown on the bottom right portion of the tumor, indicating a high variation in the voxel intensity in this area. It is easy to see that the hot spots in the original PET images and the heterogeneity images are quite different in terms of both size and location. This is reasonable because the parametric images are calculated based on the heterogeneity indices and therefore represent the spatial variation of tracer uptake. Efforts have been reported to use heterogeneity parametric images for tumor delineation and radiation targeting [40]. We believe such heterogeneity images could do much more than simply determining the tumor contour. One potential role for heterogeneity images in particular, we believe, is to serve as the guide for dose-boosting techniques for targeting the radiation dose at the most aggressive areas. Further validation must be done to verify the relationship between heterogeneity images and local aggressiveness of the tumor. This could be achieved by comparing the heterogeneity images to the whole-mount pathology or immunohistochemistry data. Since we do not have appropriate data to study the relationship between heterogeneity images and the distribution of aggressive colonies, we hope that CGITA may encourage investigators who own such data to test this hypothesis for expanding the use of heterogeneity indices.

We would also like to comment that, although CGITA is targeted at oncological applications, it can also be applied in other fields, such as neurology. It is also not limited to PET, as long as the images are stored in the DICOM format that CGITA can import. This makes CGITA also a useful tool for analyzing experimental animal data. We have tested CGITA with CT and MR images for heterogeneity index computation, as shown in Supplemental Figures 1 and 2 (see Supplementary Materials available online at http://dx.doi.org/10.1155/2014/248505). Quite a few CGITA features can be further improved in the future for image display, segmentation, and acceleration for computation. New texture analysis methods, such as those based on wavelets [41], will be investigated and added to CGITA in the future. At this moment, CGITA is solely for research purposes and shall not be used for clinical diagnosis. Interested developers are welcome to join the project to advance the functionalities of CGITA.

## 5. Conclusion

We present the CGITA software package for quantifying the tumor heterogeneity with molecular images. As a user-friendly, open-source program that is free for academic use, CGITA could assist investigators to apply heterogeneity analysis to their data. With a pilot cohort of eighteen advanced ORC patients treated with definitive radiotherapy, we found that heterogeneity indices may serve as better prognosis predictors for patient outcome by improving both the sensitivity and specificity. We believe that CGITA will facilitate and accelerate our understanding of the usefulness of heterogeneity quantification and its future clinical role in patient management. Furthermore, we hope an open-source software model like CGITA can facilitate the establishment of clinical standards for heterogeneity analysis in the future to further expand its clinical use.

## Supplementary Material

Supplementary figures show the screen shot of CGITA when CT and MRI images are loaded into the software, demonstrating that CGITA is capable of processing images from other modalities and using those images to compute heterogeneity indices.

## Figures and Tables

**Table 1:** Summary of the currently supported heterogeneity indices of CGITA.

Parent matrix	Feature measure
Cooccurrence matrix [17]	Second angular moment, contrast, entropy, homogeneity, dissimilarity, inverse difference moment
Voxel-alignment matrix [19]	Short-run emphasis, long-run emphasis, intensity variability, run-length variability, run percentage, low-intensity run emphasis, high-intensity run emphasis, low-intensity short-run emphasis, high-intensity short-run emphasis, low-intensity long-run emphasis, high-intensity long-run emphasis
Neighborhood intensity difference matrix [20]	Coarseness, contrast, busyness, complexity, strength
Intensity size-zone matrix [21]	Short-zone emphasis, large-zone emphasis, intensity variability, size-zone variability, zone percentage, low-intensity zone emphasis, high-intensity zone emphasis, low-intensity short-zone emphasis, high-intensity short-zone emphasis, low-intensity large-zone emphasis, high-intensity large-zone emphasis
Normalized cooccurrence matrix [17]	Second angular moment, contrast, entropy, homogeneity, inverse difference moment, dissimilarity, correlation
Voxel statistics	Minimum SUV, maximum SUV, mean SUV, SUV variance, SUV SD, SUV skewness, SUV kurtosis, SUV skewness (bias corrected), SUV kurtosis (bias corrected), TLG, tumor volume, entropy, SUL_peak_
Texture spectrum [15]	Max spectrum, Black-white symmetry
Texture feature coding [16]	Coarseness, homogeneity, mean convergence
Texture feature coding cooccurrence matrix [16]	Second angular moment, contrast, entropy, homogeneity, intensity, inverse difference moment, correlation, variance, code similarity
Neighborhood gray-level dependence [22]	Small-number emphasis, large-number emphasis, number nonuniformity, second moment, entropy

**Table 2:** Summary of the software features of CGITA.

Feature	CGITA implementation
Programming environment	MATLAB (MathWorks Inc.)
License	Free for academic use
Source code availability	Open source
Supported image format	DICOM (either local files or direct access to a PACS server for image retrieval)
Supported VOI format	DICOM-RT, PMOD
Currently supported textural features	72
Other features	(i) Parametric imaging of heterogeneity indices (ii) Batch mode for processing a large cohort (iii) Compiled stand-alone application available (iv) Supporting user-defined functions for heterogeneity calculation

**Table 3:** Comparison of AUC, specificity, and sensitivity of heterogeneity indices vs. SUV_mean_ and TLG.

Parent	Feature	AUC	Sensitivity (%)	Specificity (%)	*P* value^†^
Intensity-size-zone	Low-intensity short-zone emphasis	0.90*	77.8	88.9	0.004
Intensity-size-zone	Short-zone emphasis	0.81*	77.8	66.7	0.024
Texture Feature Coding Cooccurrence	Contrast	0.72	55.6	88.9	0.085
Intensity-size-zone	High-intensity zone emphasis	0.70	66.7	77.8	0.145
Intensity-size-zone	Zone percentage	0.70	55.6	88.9	0.122
SUV statistics	Entropy	0.70	66.7	77.8	0.145
SUV statistics	Mean SUV	0.60	66.7	66.7	0.453
SUV statistics	Maximum SUV	0.57	66.7	66.7	0.627
SUV statistics	TLG	0.52	55.6	66.7	0.895

*denotes that the *P* value is less than 0.05 given the null hypothesis of AUC <0.5.

^†^calculated using the Kruskal-Wallis test (19 indices have a *P* value greater than 0.453).

## References

[1] Denison TA, Bae YH (2012). Tumor heterogeneity and its implication for drug delivery. *Journal of Controlled Release*.

[2] Longo DL (2012). Tumor heterogeneity and personalized medicine. *The New England Journal of Medicine*.

[3] Shibata D (2012). Heterogeneity and tumor history. *Science*.

[4] Cook GJ, Yip C, Siddique M (2013). Are pretreatment 18F-FDG PET tumor textural features in non-small cell lung cancer associated with response and survival after chemoradiotherapy?. *Journal of Nuclear Medicine*.

[5] Huang B, Chan T, Kwong DL, Chan WK, Khong PL (2012). Nasopharyngeal carcinoma: investigation of intratumoral heterogeneity with FDG PET/CT. *American Journal of Roentgenology*.

[6] Kidd EA, Grigsby PW (2008). Intratumoral metabolic heterogeneity of cervical cancer. *Clinical Cancer Research*.

[7] Kumar V, Gu Y, Basu S (2012). Radiomics: the process and the challenges. *Magnetic Resonance Imaging*.

[8] Lambin P, Rios-Velazquez E, Leijenaar R (2012). Radiomics: extracting more information from medical images using advanced feature analysis. *European Journal of Cancer*.

[9] Salamon J, Derlin T, Bannas P (2013). Evaluation of intratumoural heterogeneity on (18)F-FDG PET/CT for characterization of peripheral nerve sheath tumours in neurofibromatosis type 1. *European Journal of Nuclear Medicine and Molecular Imaging*.

[10] Tixier F, Cheze le Rest C, Hatt M (2011). Intratumor heterogeneity characterized by textural features on baseline18F-FDG PET images predicts response to concomitant radiochemotherapy in esophageal cancer. *Journal of Nuclear Medicine*.

[11] Visvikis D, Hatt M, Tixier F, Cheze le Rest C (2012). The age of reason for FDG PET image-derived indices. *European Journal of Nuclear Medicine and Molecular Imaging*.

[12] Watabe T, Tatsumi M, Watabe H (2012). Intratumoral heterogeneity of F-18 FDG uptake differentiates between gastrointestinal stromal tumors and abdominal malignant lymphomas on PET/CT. *Annals of Nuclear Medicine*.

[13] Chicklore S, Goh V, Siddique M, Roy A, Marsden PK, Cook GJ (2013). Quantifying tumour heterogeneity in (18)F-FDG PET/CT imaging by texture analysis. *European Journal of Nuclear Medicine and Molecular Imaging*.

[14] Eichelberg M, Riesmeier J, Wilkens T, Hewett AJ, Barth A, Jensch P Ten years of medical imaging standardization and prototypical implementation: the DICOM standard and the OFFIS DICOM Toolkit (DCMTK).

[15] He D-C, Wang L (1991). Texture features based on texture spectrum. *Pattern Recognition*.

[16] Horng M-H, Sun Y-N, Lin X-Z (2002). Texture feature coding method for classification of liver sonography. *Computerized Medical Imaging and Graphics*.

[17] Haralick RM, Shanmugam K, Dinstein I (1973). Textural features for image classification. *IEEE Transactions on Systems, Man and Cybernetics*.

[18] Sun Y, Lu J, Yahagi T (2006). Texture classification for liver tissues from ultrasonic B-scan images using testified PNN. *IEICE Transactions on Information and Systems*.

[19] Loh H-H, Leu J-G, Luo RC (1988). The analysis of natural textures using run length features. *IEEE Transactions on Industrial Electronics and Control Instrumentation*.

[20] Amadasun M, King R (1989). Textural features corresponding to textural properties. *IEEE Transactions on Systems, Man and Cybernetics*.

[21] Thibault G, Fertil B, Navarro C Texture indexes and gray level size zone matrix: application to cell nuclei classification.

[22] Sun C, Wee WG (1983). Neighboring gray level dependence matrix for texture classification. *Computer Vision, Graphics and Image Processing*.

[23] Wahl RL, Jacene H, Kasamon Y, Lodge MA (2009). From RECIST to PERCIST: evolving considerations for PET response criteria in solid tumors. *Journal of Nuclear Medicine*.

[24] Kemp BJ, Kim C, Williams JJ, Ganin A, Lowe VJ (2006). NEMA NU 2-2001 performance measurements of an LYSO-based PET/CT system in 2D and 3D acquisition modes. *Journal of Nuclear Medicine*.

[25] Chung MK, Jeong H-S, Sang GP (2009). Metabolic tumor volume of [18F]-fluorodeoxyglucose positron emission tomography/computed tomography predicts short-term outcome to radiotherapy with or without chemotherapy in pharyngeal cancer. *Clinical Cancer Research*.

[26] Kruskal WH, Wallis WA (1952). Use of ranks in one-criterion variance analysis. *Journal of the American Statistical Association*.

[27] Dong X, Xing L, Wu P (2013). Three-dimensional positron emission tomography image texture analysis of esophageal squamous cell carcinoma: relationship between tumor 18F-fluorodeoxyglucose uptake heterogeneity, maximum standardized uptake value, and tumor stage. *Nuclear Medicine Communications*.

[28] Blouin S, Moreau MF, Baslé MF, Chappard D (2006). Relations between radiograph texture analysis and microcomputed tomography in two rat models of bone metastases. *Cells Tissues Organs*.

[29] Dominietto M, Lehmann S, Keist R, Rudin M (2013). Pattern analysis accounts for heterogeneity observed in MRI studies of tumor angiogenesis. *Magnetic Resonance in Medicine*.

[30] Risse F, Pesic J, Young S, Olsson LE (2012). A texture analysis approach to quantify ventilation changes in hyperpolarised3He MRI of the rat lung in an asthma model. *NMR in Biomedicine*.

[31] Barajas RF, Hodgson JG, Chang JS (2010). Glioblastoma multiforme regional genetic and cellular expression patterns: influence on anatomic and physiologic MR imaging. *Radiology*.

[32] Muzic RF, Cornelius S (2001). COMKAT: compartment model kinetic analysis tool. *Journal of Nuclear Medicine*.

[33] Deasy JO, Blanco AI, Clark VH (2003). CERR: a computational environment for radiotherapy research. *Medical Physics*.

[34] Friston KJ, Ashburner JT, Kiebel SJ, Nichols TE, Penny WD (2011). *Statistical Parametric Mapping: The Analysis of Functional Brain Images*.

[35] Eary JF, O’Sullivan F, O’Sullivan J, Conrad EU (2008). Spatial heterogeneity in sarcoma 18F-FDG uptake as a predictor of patient outcome. *Journal of Nuclear Medicine*.

[36] El Naqa I, Grigsby PW, Apte A (2009). Exploring feature-based approaches in PET images for predicting cancer treatment outcomes. *Pattern Recognition*.

[37] Vaidya M, Creach KM, Frye J, Dehdashti F, Bradley JD, El Naqa I (2012). Combined PET/CT image characteristics for radiotherapy tumor response in lung cancer. *Radiotherapy and Oncology*.

[38] Jakoby BW, Bercier Y, Conti M, Casey ME, Bendriem B, Townsend DW (2011). Physical and clinical performance of the mCT time-of-flight PET/CT scanner. *Physics in Medicine and Biology*.

[39] Kruggel F, Paul JS, Gertz H-J (2008). Texture-based segmentation of diffuse lesions of the brain’s white matter. *NeuroImage*.

[40] Yu H, Caldwell C, Mah K (2009). Automated radiation targeting in head-and-neck cancer using region-based texture analysis of PET and CT images. *International Journal of Radiation Oncology Biology Physics*.

[41] Joseph S, Balakrishnan K (2011). Local binary patterns, haar wavelet features and haralick texture features for mammogram image classification using artificial neural networks. *Communications in Computer and Information Science*.

