# Supplementary material for: Development and Evaluation of an Open-Source Software Package “CGITA” for Quantifying Tumor Heterogeneity with Molecular Images
Source: Biomed Res Int. 2014 Mar 17;2014:248505. doi: 10.1155/2014/248505 (PMC3976812; doi:10.1155/2014/248505)
Supplement: Supplementary file 1 — Supplementary figures show the screen shot of CGITA when CT and MRI images are loaded into the software, demonstrating that CGITA is capable of processing images from other modalities and using those images to compute heterogeneity indices. [file 248505.f1.pdf]

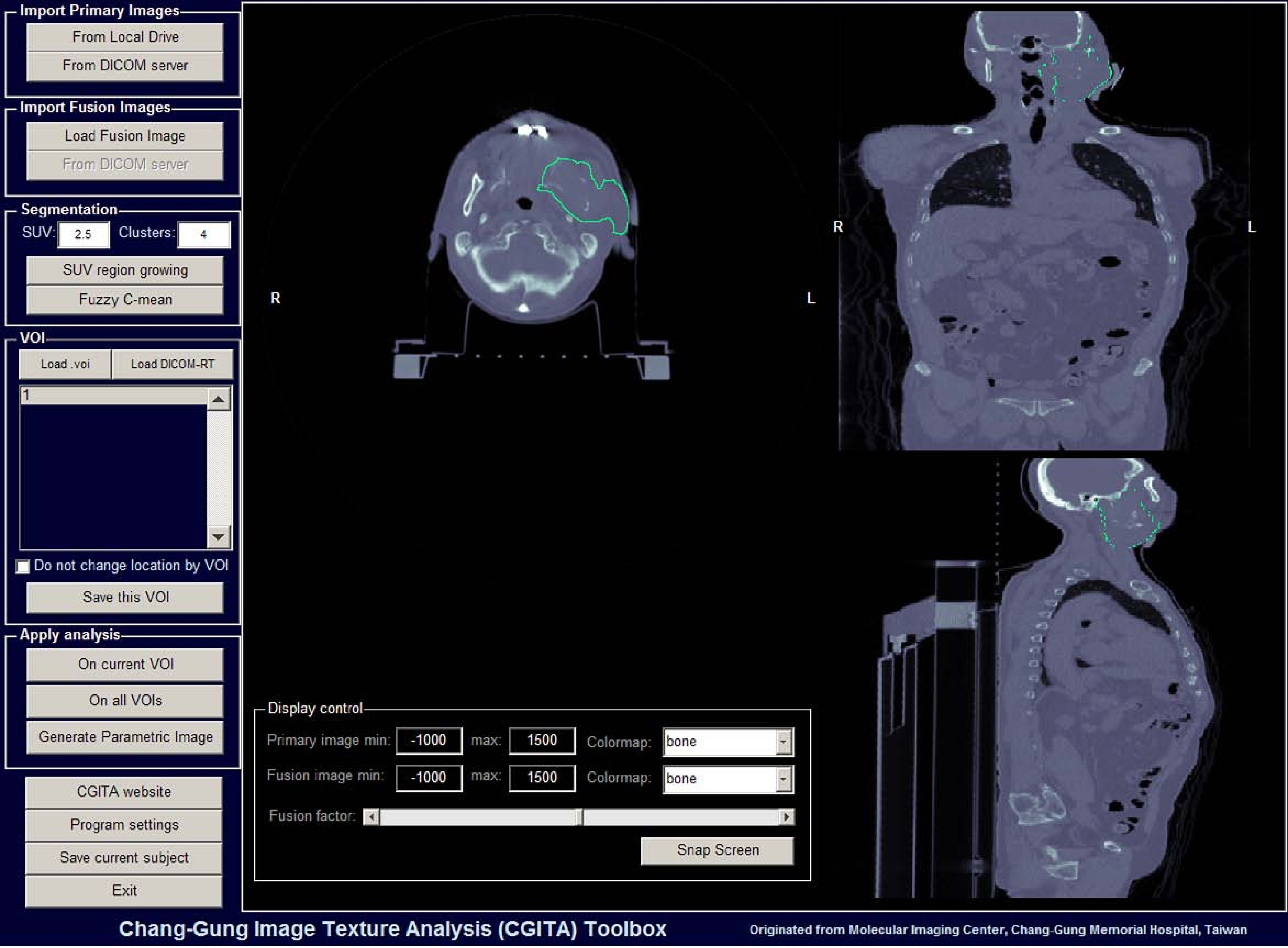

Supplemental Figure 1. Screen capture of CGITA, in which a set of patient's CT images are loaded and used for computation of heterogeneity indices.
