# Supplementary material for: Development and Evaluation of an Open-Source Software Package “CGITA” for Quantifying Tumor Heterogeneity with Molecular Images
Source: Biomed Res Int. 2014 Mar 17;2014:248505. doi: 10.1155/2014/248505 (PMC3976812; doi:10.1155/2014/248505)
Supplement: Supplementary file 2 [file 248505.f2.pdf]

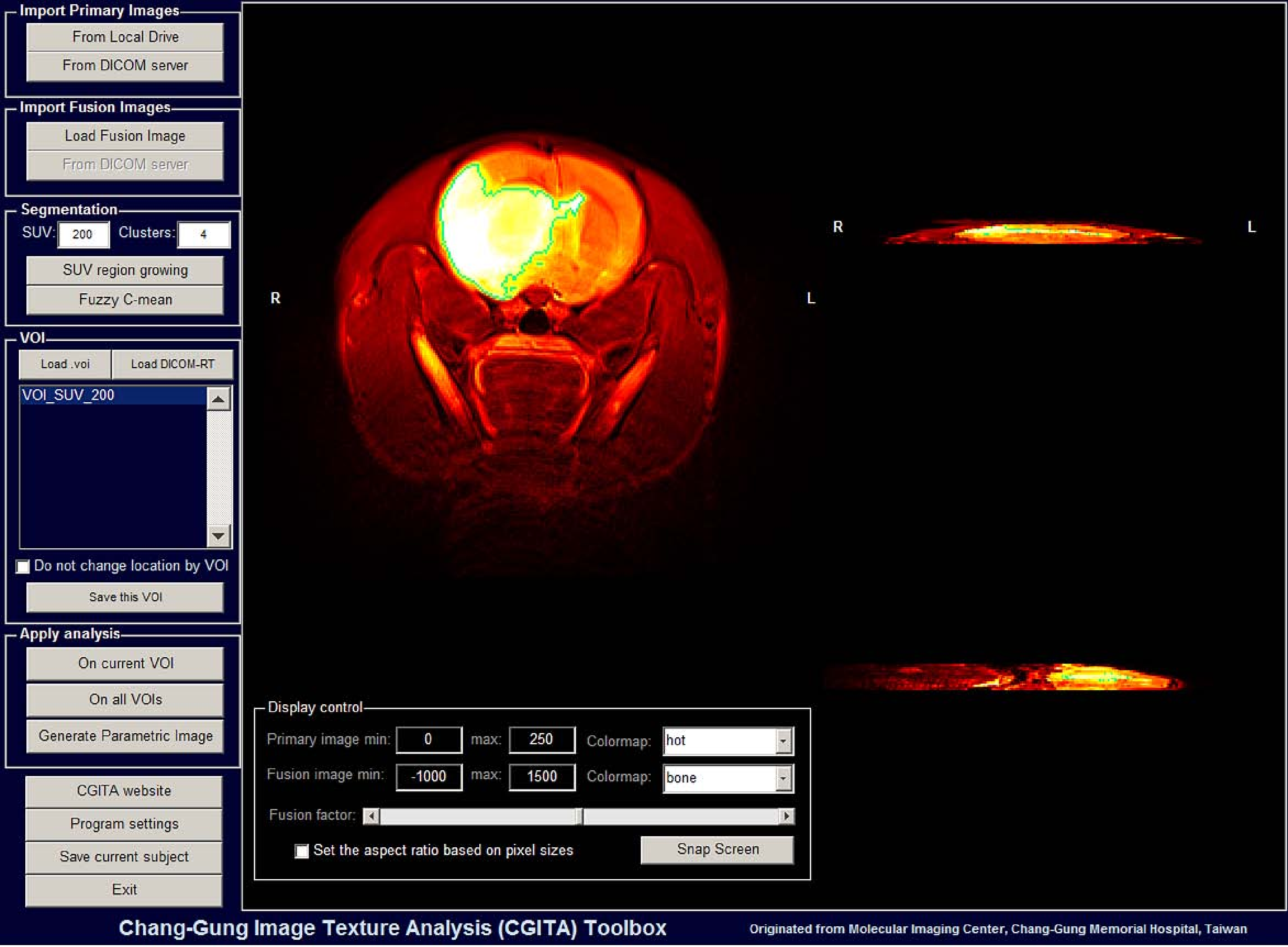

Supplemental Figure 2. Screen capture of CGITA, in which a set of animal MRI brain images are loaded and used for computation of heterogeneity indices.
